# Functional Investigation of a Novel PIWIL4 Mutation in Nonobstructive Azoospermia During the First Wave of Spermatogenesis

**DOI:** 10.3390/biom15020297

**Published:** 2025-02-17

**Authors:** Xiayu Wang, Qian Du, Wanqian Li, Zhongyu Zou, Chikun Wang, Yan Zhou, Zhibin Hu, Yayun Gu, Feng Li

**Affiliations:** 1 State Key Laboratory of Reproductive Medicine and Offspring Health, Nanjing Medical University, Nanjing 211166, China; 2021110159@stu.njmu.edu.cn (X.W.); dq@stu.njmu.edu.cn (Q.D.); liwanqian@stu.njmu.edu.cn (W.L.); zhongyuzou@njmu.edu.cn (Z.Z.); wck@stu.njmu.edu.cn (C.W.); yanzhou@njmu.edu.cn (Y.Z.); zhibin_hu@njmu.edu.cn (Z.H.); 2 Department of Epidemiology and Biostatistics, School of Public Health, Nanjing Medical University, Nanjing 210029, China; 3 Department of Urology, The Second Affiliated Hospital of Nanjing Medical University, Nanjing 210008, China

**Keywords:** PIWIL4, LINE-1, infertility, missense mutation, spermatogenesis, retrotransposon, DNA methylation

## Abstract

PIWI-interacting RNAs (piRNAs) are small noncoding RNAs that are almost exclusively expressed in germ cells to silence harmful transposons to maintain genome stability. PIWIL4 is guided by its associated piRNAs to transposable elements, where it recruits the DNA methylation apparatus and instructs de novo DNA methylation. Herein, we identified a missense variant of *PIWIL4* (c.805 C>T p.R269W) in two infertile males. Homozygous male mice carrying the orthologous knock-in variant displayed elevated transposable element expression and aberrant gene expression during the first wave of spermatogenesis, despite exhibiting normal sperm counts and morphology. Mechanistically, the mutated site altered the piRNA-binding ability of PIWIL4 and led to the derepression of endogenous LINE-1 elements. In summary, we identified a piRNA binding mutation in PIWIL4 that may be involved in human nonobstructive azoospermia.

## 1. Introduction

Infertility affects approximately 15% of couples worldwide, and males account for 50% of cases overall [1]. Male infertility can be attributed to a range of factors including testicular dysfunction, endocrinopathies, and lifestyle choices [2,3]. Currently, only approximately 4% of infertile men receive a diagnosis of genetic infertility, leaving the majority of cases classified as unexplained [4]. Spermatogenesis is an intricate and vital process in which defects can lead to abnormal sperm production, ultimately resulting in male infertility. Among the most severe forms of male infertility is nonobstructive azoospermia (NOA), characterized by a complete absence of sperm in the semen due to issues within the testes. While recent studies have identified increasing monogenic causes of NOA, our understanding of the complete spectrum of its causes remains limited [4].

PIWI-interacting RNAs (piRNAs) are small noncoding RNAs that play a crucial role in spermatogenesis [5,6,7,8]. Their significance lies in guiding PIWI-clade Argonaute (PIWI) proteins to silence transposable elements and regulate the expression of mRNAs during spermatogenesis [9]. The biogenesis and function of piRNAs involve various proteins, and mutations in PNLDC1, MOV10L1, PIWIL1, PIWIL2, and many others have been identified as causes of male infertility [10,11,12,13,14,15,16,17,18,19].

In mice, the PIWI family protein MIWI2 is expressed in male gonocytes until soon after birth [20]. During this developmental stage, the majority of piRNAs expressed in the gonocytes correspond to transposon elements [21,22]. It has been proposed that piRNAs guide nuclear PIWIL4 to transposable elements, where it recruits the DNA methylation apparatus and instructs de novo DNA methylation [20,23,24,25]. Despite these proposals, the underlying mechanisms have remained largely unexplored, leaving room for further investigation. Recently, a single-cell RNA-Seq analysis of patients with impaired spermatogenesis revealed intriguing findings. An increase in the number of the most undifferentiated spermatogonia (PIWIL4+) and a reduction in the number of Adark reserve stem cells were observed [26]. Additionally, PIWIL4+ spermatogonia are crucial for efficient testis regeneration during stress [27]. However, whether mutations in PIWIL4 affect spermatogonial differentiation and spermatogenesis in humans remains unknown, highlighting the need for future research in this area.

We conducted Sanger sequencing of 620 infertile men with nonobstructive azoospermia and identified potential harmful mutations in the *PIWIL4* gene. Notably, a heterozygous missense variant of *PIWIL4* (c.805 C>T p.R269W) was found in two infertile men from unrelated families. To further investigate the implications of this variant, we generated a mouse model with an orthologous missense mutation in *Piwil4* using the CRISPR-Cas9 gene editing method. Surprisingly, while heterozygous and homozygous *Piwil4* knock-in (KI) male mice exhibited normal sperm counts and morphology, they displayed an increased expression of retrotransposon elements and abnormal gene expression during the first wave of spermatogenesis. To our knowledge, there has been limited research on the function of *PIWIL4* in human infertility, and our study provides the first evidence that the identified missense mutation in *PIWIL4* may lead to male infertility in a manner distinct from the effects observed in the mouse model.

## 2. Materials and Methods

### 2.1. Ethics Statement

All animal procedures and experiments were approved by the Institutional Animal Care and Use Committee of Nanjing Medical University (IACUC-2108032). The human study was approved by the research ethics committee of Nanjing Medical University. All patients provided written informed consent before taking part in this research.

### 2.2. NOA Patient Population

A total of 620 patients with nonobstructive azoospermia and 2713 fertile males were recruited at the Clinical Center of Reproductive Medicine in Nanjing for this population study. All control males had fathered at least one healthy child. All participants signed an informed consent document. Semen sperm analysis was performed in biological replicates to confirm the absence of sperm, and patients with a medical history of cryptorchidism, testicular trauma, vasectomy procedure, orchitis, obstruction of the vas deferens, testis inflammation, or endocrine disorders were excluded. These azoospermia patients were further selected to exclude the most common genetic causes of infertility including chromosomal abnormalities and Y chromosomal microdeletions. A 5-mL sample of whole blood was obtained from each participant as a source of genomic DNA for further Sanger sequencing analysis.

### 2.3. Generation of the Piwil4^R264W/R264W^ Knock-In Mice Model

All animal experiments were approved by the Institutional Animal Care and Use Committee of Nanjing Medical University and were performed in accordance with the guidelines and regulations of the committee. The knock-in mice were generated by CRISPR-Cas9 technology. sgRNAs and donor ssDNA were microinjected into the cytoplasm of C57BL/6 mouse zygotes.

### 2.4. Histological Analysis and Immunohistochemistry

Testes and epididymides were freshly fixed in Bouin’s solution (Sigma, Missouri, USA) overnight at room temperature and dehydrated with ethanol (30%, 50%, 70%, 95%, 100%). Then, the samples were embedded in paraffin. Sections (5 μm thick) were prepared and stained with hematoxylin and eosin. For immunohistochemistry, testes were fixed in 4% paraformaldehyde (PFA), embedded in paraffin, and sliced into 5 μm sections. After deparaffinization, rehydration, and microwave antigen retrieval, the slides were incubated with 3% H_2_O_2_ in methyl alcohol for 10 min. The sections were incubated with 5-methylcytosine (Active Motif, California, USA) antibody at a 1:200 dilution at 4 °C overnight. Then, the slides were incubated with secondary antibody at room temperature for 30 min, stained with DAB substrate for 7 min, and sealed after dehydration.

### 2.5. Immunofluorescence and TUNEL Assay

For the immunofluorescence assay, testes were fixed in 4% paraformaldehyde (PFA) overnight at 4 °C. Subsequently, the testes were dehydrated with ethanol (70%, 95%, 100%), immersed in xylene, embedded in paraffin, and cut into 5 μm sections. Sections were dewaxed in xylene twice, followed by dehydration in ethanol (100%, 95%, 70%, 50%, 0%). The slides were boiled in sodium citrate (Servicebio, Wuhan, China) antigen retrieval solution for 15 min, washed three times with PBS, blocked with blocking buffer (10% FBS, 1% Triton X-100, 1% BSA in PBS) for 1 h at room temperature, and incubated with primary antibodies at 4 °C overnight. This was followed by incubation with secondary antibodies for 1 h, after which the samples were blocked with Antifade Mounting Medium (Beyotime, Shanghai, China). For the TUNEL assay, the sections were analyzed with a TUNEL Enzyme and Label Kit (Roche Boehringer Mannheim, Mannheim, Germany). All staining of testis sections was visualized on a confocal microscope (Carl Zeiss, **Oberkochen, Germany**).

### 2.6. Plasmids and Cell Transfection

p3×Flag-PIWIL4 and p3×Flag-PIWIL4^R264W/R264W^ were constructed through the insertion of cDNAs into the p3×Flag-CMV-10 (Sigma) plasmid. The PIWIL4 mutant was constructed using plasmid and KOD-Plus-Mutagenesis Kits (Toyobo). Chemically synthesized piRNAs and plasmids were transfected into HEK293T cells with Lipofectamine 3000 (Invitrogen, **California, USA**) according to the manufacturer’s protocol. Cells were collected 36–48 h after transfection.

### 2.7. Real-Time Quantitative PCR (RT–qPCR) Analyses and RNA-seq

The total RNA from mouse testes was isolated by utilizing TRIzol reagent (Invitrogen, California, USA). The concentration and purity of RNA were determined by measuring the absorbance at 260/280 nm. Total RNA was reverse transcribed into complementary DNA (cDNA) by PrimeScript RT Master Mix (Takara, Kusatsu, Japan). Real-time qPCR was conducted by utilizing SYBR Premix Ex Taq II (Vazyme, Nanjing, China) on an iCycler RT-PCR Detection System (ABI). For data analysis, the comparative CT (ΔΔCT) method was used to calculate the relative quantification of gene expression. For the quantification of retrotransposons, primers were designed according to a previous study [28]. For RNA-Seq, sequencing libraries were generated using the VAHTS mRNA-seq v2 Library Prep Kit for Illumina following the manufacturer’s recommendations, and index codes were added to attribute sequences to each sample. The libraries were sequenced on an Illumina NovaSeq platform to generate 150 bp paired-end reads. According to the manufacturer’s instructions, to obtain high-quality clean reads, the reads were further filtered by fastp (version 0.18.0). The mapped reads of each sample were assembled by using StringTie v1.3.1 via a reference-based approach. For each transcription region, an FPKM (fragment per kilobase of transcript per million mapped reads) value was calculated to quantify its expression abundance and variations using String Tie software (version 1.3.1 Maryland, USA). RNA differential expression analysis was performed by DESeq2 software (version 1.3.1 Heidelberg, Germany) between two different groups (and by edgeR between two samples). Genes/transcripts with a false discovery rate (FDR) below 0.05 and an absolute fold change ≥2 were considered differentially expressed genes/transcripts.

### 2.8. Immunoblot Analysis

The proteins from the cultured cells and mouse testes tissues were lysed in lysis buffer containing a protease inhibitor cocktail. The lysate was placed on a shaker and rotated for one hour. Proteins were separated by electrophoresis on 8% SDS-polyacrylamide gels and then transferred onto polyvinylidene difluoride (PVDF) membranes (Millipore, **Massachusetts, USA**) for immunoblot analysis. Details of the antibodies used for Western blotting, co-IP, and immunofluorescence staining can be found in the Supplementary Materials.

### 2.9. CASA

Following euthanasia of the mice, the caudal epididymis was immediately excised. Two incisions were made on the epididymal tail, which was then placed in 350 μL HTF medium (Irvine Scientific, Santa Ana, CA, USA) containing 10% fetal bovine serum for 5 min at 37 °C. Hamilton Thorne’s Ceros II system (Beverly, MA, USA) was used for the measurement of sperm motility.

### 2.10. In Vitro Fertilization (IVF)

Two-month-old C57BL/6 wild-type female mice were intraperitoneally injected with 6 units of pregnant mare serum gonadotropin (PMSG), followed by the injection of 6 units of human chorionic gonadotropin (hCG) 48 h later to induce ovulation. Following 13 h of ovulation induction, in vitro fertilization (IVF) experiments were conducted. Sperm samples from wild-type, heterozygous, and homozygous mice were collected from the epididymis and incubated in HTF medium for one hour to allow capacitation. After 13 h of ovulation induction, cumulus-intact oocytes collected from superovulated females were transferred to an HTF medium drop. Then, 10 μL of the sperm solution was added to the HTF medium containing the collected oocytes. After incubating for 5 h, the embryos were washed in HTF drops and transferred to KSOM culture media for further cultivation at 37 °C under 5% CO_2_. Fertilization rates were assessed by counting two-cell embryos at 20 h postfertilization.

### 2.11. RIP-Seq

In brief, cells and testes were lysed in lysis buffer [100 mM KCl,5 mM MgCl_2_, 10 mM HEPES, 0.5% NP-40 containing 10 U/mL]. RNase inhibitor (Takara, Kusatsu, Japan) and a protease inhibitor cocktail (Roche, Basel, Switzerland) were used. After centrifugation, the supernatants were precleared for 1 h at 4 °C with protein A beads. Protein A beads were incubated with anti-Flag or anti-PIWIL4 at 4 °C overnight. For immunoprecipitation, 1 mL of lysate was incubated with 100 μL of antibody-bound beads with rotation for 4 h at 4 °C. The bead complexes were then washed five times with washing buffer [10 mM Tris-HCl (pH 8.0), 150 mM NaCl, 0.01% NP-40, 1 mM MgCl_2_, and 5% glycerol]. The immunoprecipitates and input samples were treated with proteinase K before RNA extraction. For qPCR analysis, immunoprecipitated RNA was reverse-transcribed using PrimeScript RT Master Mix (TaKaRa, Kusatsu, Japan).

### 2.12. Bisulfite Conversion and PCR

Genomic DNA was extracted using the TIANamp Genomic DNA Kit (DP304-02). Bisulfite conversion of 500 ng of genomic DNA was conducted using the EZ DNA Methylation-Gold™ Kit (Zymo Research, D5006, Irvine, CA, USA). Regions of interest were amplified by PCR using Premix Ex Taq™ Hot Start Version DNA Polymerase (Takara, Kusatsu, Japan). For Sanger sequencing, PCR products were extracted from 1% agarose gels and cloned and inserted into the pESI-T plasmid for sequencing. The sequences were analyzed and aligned by QUMA software (version 1.0.0 Fukuoka, Japan) [29].

### 2.13. Testicular Germ Cell Depletion and Regeneration

Busulfan ( Selleck, **Texas, USA**) was prepared according to the manufacturer’s instructions. A single dose (30 mg/kg) was administered via i.p. injection. After 8 weeks, the mice were euthanized and weighed. Both testes were collected and weighed. The percentage of regenerated seminiferous tubules containing germ cells up to the spermatid stage was quantified in H&E-stained testis sections.

## 3. Results

### 3.1. Identification of a Heterozygous Missense PIWIL4 Variant in Two Men from Unrelated Families with Nonobstructive Azoospermia

**Figure 1:**
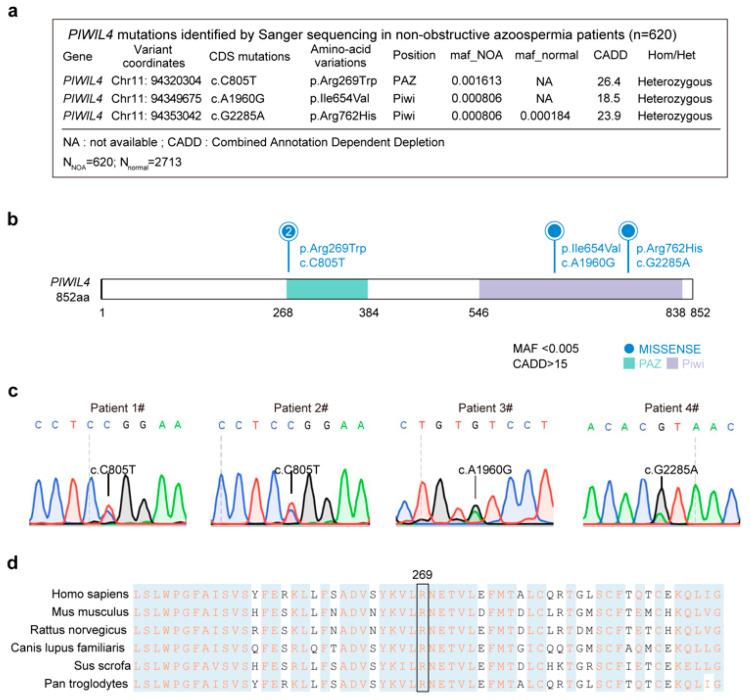
A heterozygous PIWIL4 mutation identified in two unrelated Chinese patients with NOA. (**a**) *PIWIL4* mutations identified in four patients with nonobstructive azoospermia. Genomic DNA samples were isolated from peripheral blood samples of these azoospermic patients and subjected to Sanger sequencing. Three heterozygous missense mutations were identified: a heterozygous missense mutation (c.805C>T [p.R269W]), a heterozygous missense mutation (c.1960A>G [p.I654V]), and a heterozygous missense mutation (c.2285G>A [p.R762H]). Genomic variants were screened using the following criteria: minor allele frequency (MAF) < 0.005 and CADD score >15. (**b**) The PIWIL4 protein consists of a PAZ domain, which is responsible for anchoring the 3′ ends of small RNAs, and a PIWI domain structurally similar to RNase H. In the human PIWIL4 protein, one mutation is located in the PAZ domain, while the other three are located in the PIWI domain. The missense mutation c.805 C>T leads to an amino acid substitution from arginine to tryptophan. (**c**) Sequence alignment showing the conservation of the mutated amino acid across different species. (**d**) Sanger sequencing confirmed four heterozygous missense mutations in azoospermia patients.

To identify *PIWIL4*-associated mutations associated with human male infertility, we conducted Sanger sequencing of the 20 exons and intron boundaries of the *PIWIL4* gene from a cohort of 620 NOA patients. Semen analyses were performed in biological duplicates for all patients, and those diagnosed with orchitis, obstruction of the vas deferens, or endocrine disorders were excluded. Single nucleotide variants (SNVs) were screened based on the criterion that the minor allele frequency (MAF) was <0.005 in the gnomAD database, and their deleteriousness was predicted using the Combined Annotation Dependent Depletion (CADD) tool with a score > 15 (Figure 1a).

PIWIL4 is composed of a PAZ domain, which is responsible for anchoring the 3′ ends of small RNAs, and a PIWI domain structurally similar to RNase H (Figure 1b). We identified three unique (c.805 C>T; c.1960A>G; c.2285G>A) heterozygous variants in four unrelated patients with NOA but absent in 2713 fertile controls (Figure 1a–c). The missense mutation c.805 C>T (MAF = 0 in gnomAD populations) was identified in two patients, resulting in an amino acid substitution from arginine to tryptophan. Notably, this mutation occurs at a highly conserved site across different species, particularly in mammals (Figure 1d). Furthermore, it is located within the PAZ domain, and is known to bind specifically to the 3′ ends of piRNAs [30,31].

### 3.2. Altered Expression of the LINE-1 Transposon in Piwil4^R264W/R264W^ Mutant Male Mice

We employed CRISPR-Cas9 technology to generate a knock-in mutation in the mouse ortholog of *PIWIL4*, and the genotypes of the resulting strains were confirmed by Sanger sequencing. To ensure precision, we designed two different single guide RNAs (sgRNAs) targeting the DNA sequence near the mutant site. We verified their specificity and confirmed the absence of off-target effects on known coding and noncoding genes (Figure S1). In mice, PIWIL4 exhibits a temporally regulated expression pattern that is expressed from E15.5 to P3 and thus spans the developmental window from prespermatogonia to spermatogonia [20]. Notably, PIWIL4 is expressed not only in fetal gonocytes, but also in a small population of adult spermatogonia with SSC activity, which is crucial for efficient regenerative ability following injury [27]. Of particular importance is the role of piRNAs in directing nuclear PIWIL4 to active transposable element loci, where it induces silencing through DNA methylation. Disruption of PIWIL4 function, as observed in knockout models, leads to the overexpression of LINE-1 and IAP retrotransposons [20].

**Figure 2:**
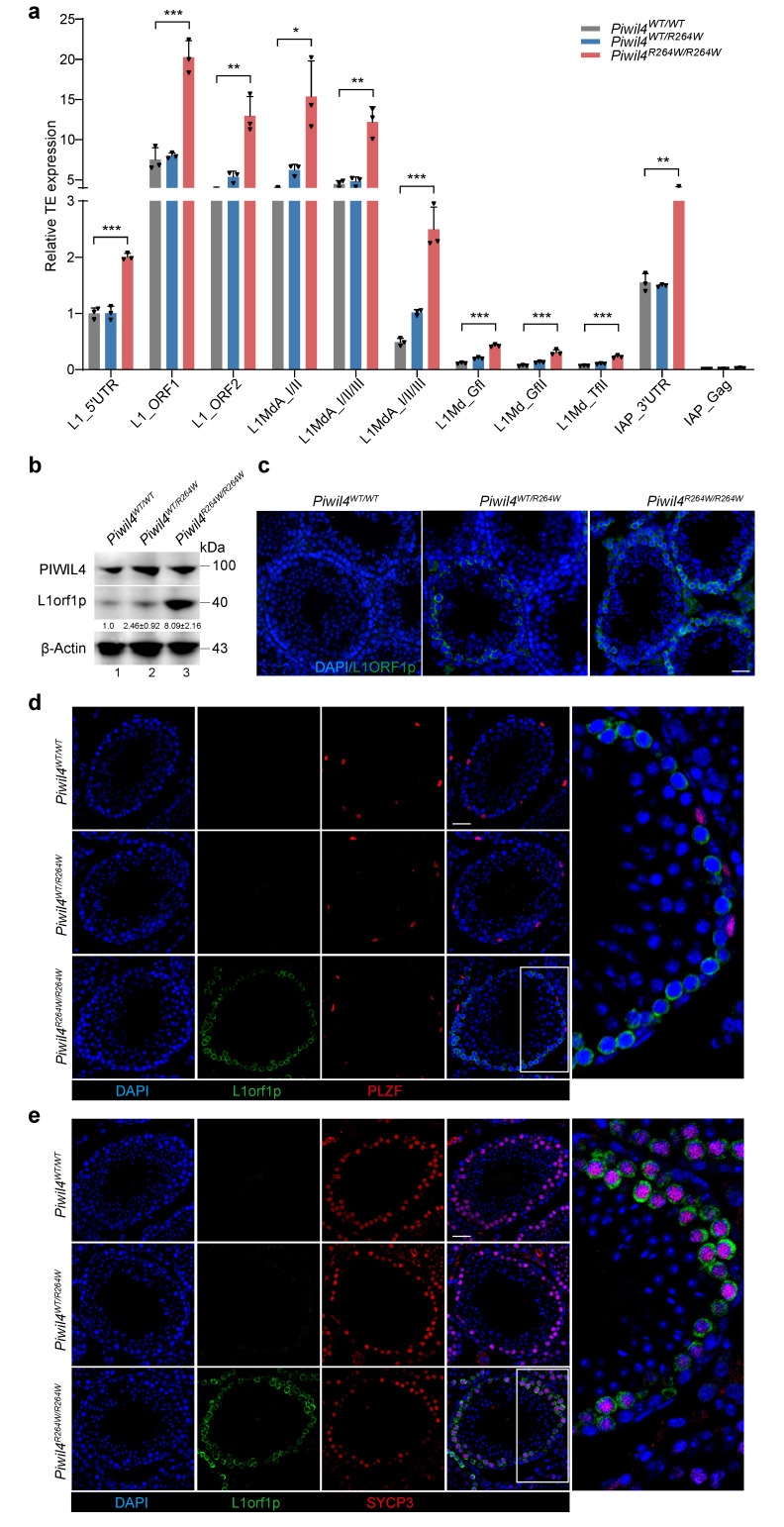
L1 expression is induced in spermatocytes in *Piwil4* knock-in male mice. (**a**) RT–qPCR analysis of LINE-1 subfamily expression in 5-week-old *Piwil4^WT/WT^*, *Piwil4^WT/R264W^*, and *Piwil4^R264W/R264W^* mice testis. (**b**,**c**) Western blot (right) and immunofluorescence (left) analyses of 5-week-old *Piwil4^WT/WT^*, *Piwil4^WT/R264W^,* and *Piwil4^R264W/R264W^* mice testis with anti-L1orf1p antibody are shown. Scale bars, 20 μm. (**d**,**e**) Immunofluorescence with anti-PLZF/SYCP3 and anti-L1orf1p antibodies in 5-week-old *Piwil4^WT/WT^*, *Piwil4^WT/R264W^,* and *Piwil4^R264W/R264W^* mice testicular sections, respectively. Scale bars, 50 μm. The results shown are representative of three independent experiments. Statistics: Student’s *t* test, * *p* < 0.05, ** *p* < 0.01, *** *p* < 0.001.Original images of (**b**) can be found in Supplementary Materials.

To investigate the function of PIWIL4 during the first wave of spermatogenesis, we examined the expression of L1orf1p (a unique LINE-1 encoded RNA-binding protein) in 5-week-old and 12-week-old mice. Surprisingly, the protein levels of L1orf1p increased in the testes of 5-week-old *Piwil4^R264W/R264W^* mice but returned to normal in 12-week-old mice (Figure 2b,c and Figure S2c,d). RT–qPCR analysis further confirmed the derepression of LINE-1 subfamilies in *Piwil4^R264W/R264W^* mice (Figure 2a). Immunofluorescence staining revealed that L1orf1p was highly expressed in spermatocytes but not in spermatogonia, as evidenced by the expression of specific markers (Figure 2d,e). Previous studies have highlighted the different effects of PIWIL2 and PIWIL4 on piRNA biogenesis and DNA methylation, with PIWIL2 being responsible for DNA methylation of a larger subset of TE families than PIWIL4 [32]. Importantly, researchers have found that LINE-1 derepression in spermatocytes may not necessarily lead to meiosis arrest [33]. Collectively, our results indicate that mutant PIWIL4 in PGCs/prospermatogonia leads to retrotransposon derepression in spermatocytes during the first wave of spermatogenesis in mice.

### 3.3. Mutant PIWIL4 Does Not Affect Normal Spermatogenesis or Sperm Morphology

The first round of mouse spermatogenesis is a distinctive process characterized by the absence of self-renewing spermatogonia stage, and PIWIL4 plays a crucial role in primordial germ cell development [34,35]. To investigate the impact of the mutation, we conducted histological analysis of 12-week-old *Piwil4^R264W/R264W^* testes (Figure S2a,b). Surprisingly, histological analyses revealed that *Piwil4^R264W/R264W^* male mice exhibited normal seminiferous tubules and spermatogenesis (Figure S2a,b). To assess sperm quality, computer-assisted sperm analysis (CASA) was performed on *Piwil4^R264W/R264W^* mice. The percentages of viable and total motile sperm in *Piwil4^R264W/R264W^* mice were within the normal range, and there were no significant differences in progressive sperm, sperm velocity (VCL), straight-line velocity (VSL), average-path velocity, or beat/cross frequency (BCF). There was also no significant difference in the apoptosis levels of the mutant mice (Figure S2e). During a breeding period of approximately 180 days, the number of pups per litter was 7.6 for the *Piwil4^R264W/R264W^* mutant males compared with 6.1 for control males with no statistically significant difference (Figure S2f). These results indicate that the mutation at the R264 site does not have a significant impact on adult mouse spermatogenesis. The breeding results suggest that *Piwil4^R264W/R264W^* males are capable of normal reproductive function, similar to those in the control group.

### 3.4. The First Wave of Spermatogenesis Is Damaged in Piwil4^R264W/R264W^ Mutant Male Mice

**Figure 3:**
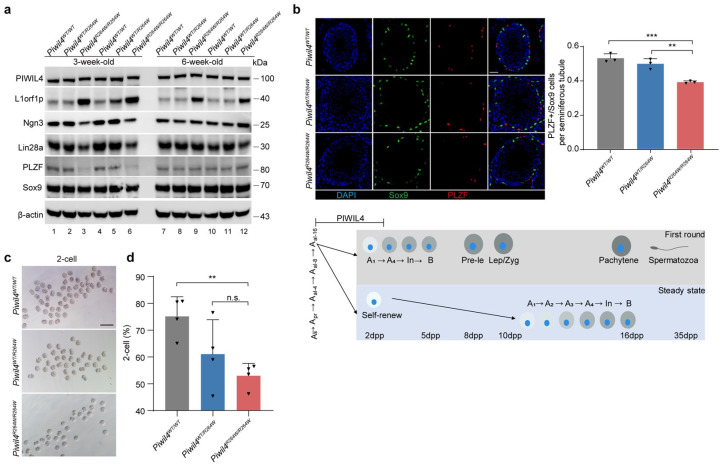
Defective proliferation of differentiating spermatogonia in the first wave of spermatogenesis in *Piwil4* knock-in mice testis. (**a**) Western blot analysis of markers for different stages of spermatogenesis in *Piwil4^WT/WT^*, *Piwil4^WT/R264W^*, and *Piwil4^R264W/R264W^* mice testis. (**b**) Immunofluorescence staining of *Piwil4^WT/WT^*, *Piwil4^WT/R264W^*, and *Piwil4^R264W/R264W^* mice testis with anti-PLZF and anti-Sox9 antibodies are shown. The numbers of PLZF^+^ spermatogonia and Sox9^+^ Sertoli cells per tubule were quantified according to the imaging results from at least 3 mice. Scale bars, 50 μm. (**c**) Top, immunofluorescence staining of *Piwil4^WT/WT^*, *Piwil4^WT/R264W^,* and *Piwil4^R264W/R264W^* mice testis with anti-LIN28A antibodies are shown. The numbers of LIN28A+ cells per tubule were quantified according to the imaging results from at least 3 mice. Scale bars, 50 μm. Bottom, schematic representation of the first wave of spermatogenesis and steady-state spermatogenesis in mice. (**d**) Representative two-cell embryos from in vitro fertilization. Scale bar, 100 μm. (**e**) The percentages of two-cell embryos were counted. Each group consisted of at least three male mice. (**f**) Sperm concentration of 6-week-old *Piwil4^WT/WT^*, *Piwil4^WT/R264W^*, and *Piwil4^R264W/R264W^* mice. (**g**) Sperm motility of 6-week-old *Piwil4^WT/WT^*, *Piwil4^WT/R264W^*, and *Piwil4^R264W/R264W^*. Each group consisted of at least three male mice. The mean ± SD of three separate experiments was plotted. Statistics: Student’s *t* test, ** *p* < 0.01, *** *p* < 0.001, n.s., not significant. Original images of (**a**) can be found in Supplementary Materials.

PIWIL4 is expressed not only in fetal gonocytes, but also in a small population of adult spermatogonia. We sought to determine whether this mutation compromised PIWIL4 function in undifferentiated or differentiating spermatogonia and to investigate how aberrant retrotransposon activity impacted the initiation of spermatogenesis. To this end, Western blotting was performed with mice testis, and the results showed that the expression of differentiating undifferentiated spermatogonial markers reduced significantly in 3-week-old testes (Figure 3a). In addition, the ratio of PLZF+/Sox9+ per tubule was reduced in mutant mice, as determined by staining for PLZF and Sox9, which are markers of undifferentiated spermatogonia and Sertoli cells, respectively (Figure 3b). We next immunostained the mice testes with LIN28A, a marker of undifferentiated spermatogonia (Figure 3c). As predicted, the number of undifferentiated (LIN28A-positive) spermatogonia was decreased in the mutant mice (Figure 3c). The reduced number of PLZF+ and LIN28A+ spermatogonia did not result in an obvious phenotypic change during the first wave of spermatogenesis in *Piwil4^R264W/R264W^* male mice (Figure S3a,b).

Considering that the first wave of spermatogenesis in mice is initiated a few days after birth and may differ from the steady state, we performed in vitro fertilization (IVF) using sperm from *Piwil4^R264W/R264W^* male mice. We collected sperm from the first wave of spermatogenesis by isolating epididymal spermatozoa from the cauda of 6-week-old male mice. Interestingly, the rate of two-cell (2-cell) formation was significantly lower in the mutant mice than in the wild-type mice (Figure 3d,e). Additionally, we examined sperm concentration and motility and observed that although there was a trend toward decreased values in mutant mice, the difference with wild-type mice did not attain significance (Figure 3f,g). In summary, sperm produced in mutant mice during the first wave of spermatogenesis display defects in fertilization capacity, correlated with the derepression of retrotransposons.

### 3.5. Mutant PIWIL4 Shows Altered piRNA Loading Ability

Initially, the expression levels of the PIWIL4 protein were examined, and no significant change was detected in the mutant testes compared with the wild-type testes (Figure S2d). PIWI proteins are known to have four conserved domains: the N-terminal, PAZ, MID, and PIWI domains. The mutant site is located in the PAZ domain, which is responsible for binding specifically to the 3′ ends of piRNAs. Since the localization of PIWIL4 in the nucleus is dependent on its associated piRNAs, we detected whether this mutation affected the entry of PIWIL4 into the nucleus. However, we found that in *Piwil4^R264W/R264W^* mice, PIWIL4 was located in the nucleus without a significant difference (Figure S4a). To rule out the possibility that altered piRNA binding was below the threshold required for PIWIL4 retention in the cytoplasm, we quantified the two most abundantly expressed piRNAs that were previously reported to associate with PIWIL4 [36]. RNA immunoprecipitation (RNA-IP) coupled with RT–qPCR analysis revealed a significant decrease in piRNA association with the mutant PIWIL4 (Figure S4b). As AlphaFold 3 can generate predictions containing proteins and RNA [37], we tried it using piR-18-197 as an example. Interestingly, the R264W mutation may alter the hydrogen bonds surrounding the 264R site (Figure S4c,d). These results indicate that the mutant protein exhibits altered piRNA binding specificity but maintains comparable overall small RNA binding capacity.

### 3.6. Genes Harboring Intact LINE-1 Sequences Are Overexpressed in Piwil4^R264W/R264W^ Male Mice

**Figure 4:**
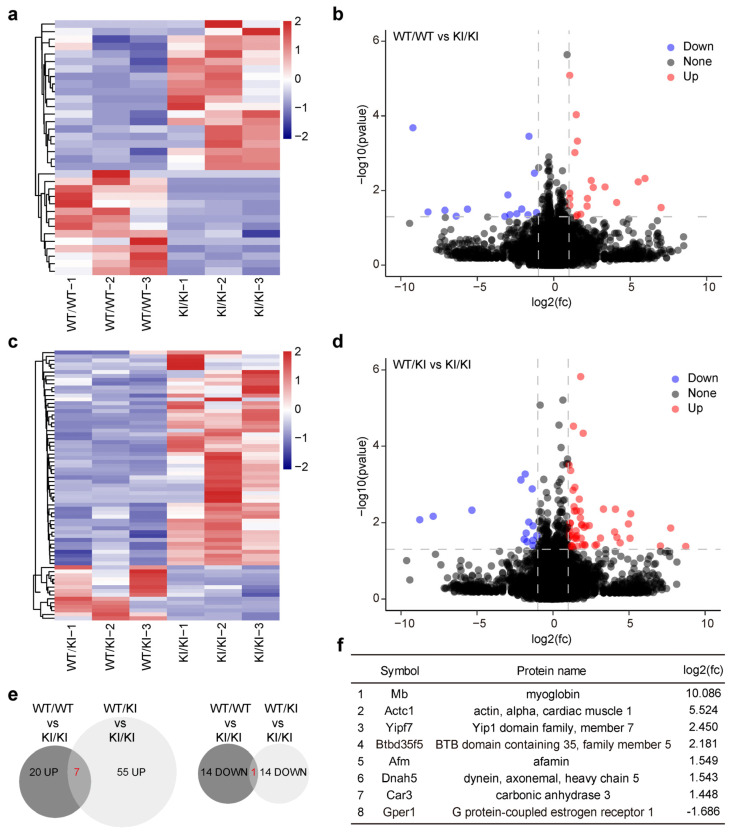
Dysregulation of transcriptomes in the testis of *Piwil4* knock-in mice. (**a**) Heatmap showing the top 34 highly differentially expressed genes (DEGs) between the testis of wild-type and homozygous knock-in mice. (**b**) Volcano plot showing the differential expression analysis of the testis of wild-type and homozygous knock-in mice. (**c**) Heatmap showing the top 69 highly differentially expressed genes (DEGs) between the testis of heterozygous and homozygous knock-in mice. (**d**) Volcano plot showing the differential expression analysis of the testis of heterozygous and homozygous knock-in mice. (**e**) Venn diagram summarizing the overlap of DEGs between the testis of the wild-type, heterozygous, and homozygous knock-in mice. Left, upregulated; right, downregulated. (**f**) The list of genes overlapping DEGs between the testes of *Piwil4^WT/WT^* vs. *Piwil4^R264W/R264W^* and *Piwil4^WT/R264W^* vs. *Piwil4^R264W/R264W^* mice.

To investigate the molecular effects of retrotransposon derepression, we performed RNA-sequencing on 3-week-old wild-type, *Piwil4^WT/R264W^*, and *Piwil4^R264W/R264W^* testes (Figure 4a–d). The data revealed the significant upregulation of 20 genes and downregulation of 14 genes in *Piwil4^R264W/R264W^* testes compared with wild-type testes, with a cutoff of >2.0-fold change and *p* < 0.05 (Figure 4e). Notably, among the upregulated genes, Afamin and Yipf7 had intact LINE-1 sequences belonging to the L1MdTf_III subfamily in their intron.

**Figure 5:**
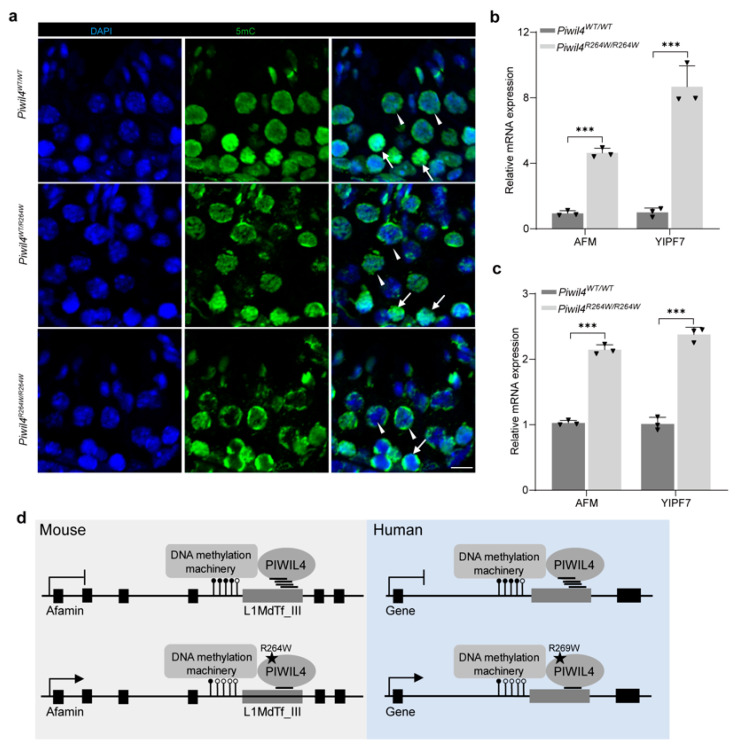
DNA hypomethylation of LINE-1 family members in *Piwil4* knock-in male mice. (**a**) Immunofluorescence staining of 5-methylcytosine (5mC) modifications in testis sections. Arrows indicate spermatocytes and arrowheads indicate round spermatids. Scale bars, 10 μm. (**b**,**c**) RT–qPCR analysis of the differentially expressed genes (DEGs) in 5-week-old and 12-week-old testis. (**d**) Working model. The mean ± SD of three mice were calculated. Statistics: Student’s *t* test, *** *p* < 0.001.

Previous studies have shown that recruitment of SPIN1–SPOCD1 through chromatin modification to young LINE1 elements is the first authentication step, and the engagement of PIWIL4/piRNA with the nascent transcript is the second licensing event [23,25]. piRNA-mediated sequence-based recognition of transposons performs DNA methylation [23,24,25]. Additionally, HUSH and MORC2 are known to bind to evolutionarily young LINE-1s and promote histone H3 Lys9 trimethylation (H3K9me3) deposition [38]. Although LINE-1 silencing in spermatogonia requires both DNA methylation and H3K9me2, DNA methylation alone can silence LINE-1 in spermatocytes [39,40]. We propose that the altered binding of piRNAs may lead to hypomethylation of L1MdTf_III subfamily elements located in the introns of Afamin and Yipf7. To test this hypothesis, we performed immunofluorescence, and the results showed reduced DNA methylation in germ cells, particularly in spermatocytes of the *Piwil4^R264W/R264W^* mice (Figure 5a). Consistent with these findings, the expression of both Afamin and Yipf7 was significantly upregulated in 5-week-old *Piwil4^R264W/R264W^* mice and partially recovered in 12-week-old mice (Figure 5b,c). Intriguingly, although human Afamin lacks intact LINE-1 sequences, it has been reported that Afamin protein levels in semen and serum are higher in patients with oligoasthenoteratozoospermia than in normal males [41].

PIWIL4 is expressed in a tiny population of adult spermatogonia with SSC activity and is essential for efficient regeneration following injury [27]. We then tested whether this mutation disrupted the function of PIWIL4 in spermatogonia amplification. To this end, we treated the mice with busulfan, which is a DNA-alkylating agent that is particularly toxic to differentiating spermatogonia. Germline regeneration is driven by undifferentiated spermatogonia that persist following busulfan treatment. However, 8 weeks after busulfan injection, the wild-type and mutant mice reached the maximum recovery with no noticeable difference (Figure S5a–c). The number of PLZF+ spermatogonia per tubule was normal compared with that in the wild-type mice (Figure S5d). These observations indicate that the regenerative capacity of undifferentiated spermatogonia remains intact in the presence of the PIWIL4 mutant.

## 4. Discussion

Our study focused on the impact of a piRNA-binding mutation in PIWIL4, a gene known to be indispensable for suppressing retrotransposons during primordial germ cell (PGC) development. In mice, PIWIL4 deficiency leads to spermatogenesis arrest at the zygotene stage of meiotic prophase I [20]. Surprisingly, despite normal fertility in both heterozygous and homozygous mutant mice, we observed derepression of retrotransposon elements specifically during the first wave of spermatogenesis. Among the significantly overexpressed genes in the mutant mice, two harbored intact L1MdTf_III in their introns. Strikingly, the expression of these genes returned to normal with age, mirroring the testicular phenotypes in mice (Figure 4b,c). However, both genes had fragmented LINE-1s in their introns and lacked the ability to mobilize autonomously in humans.

Three possible reasons may explain the observed phenotypic difference. First, although PIWIL4 expression in undifferentiated spermatogonia is conserved among humans, macaques, and mice, fundamental differences in the self-renewal and differentiation of spermatogonial stem cells exist among species. While mice lack a self-renewing spermatogonial stage during the first wave of spermatogenesis, humans maintain spermatogonia in an undifferentiated state until puberty [42]. Second, the mutation site located near the PAZ domain is critical for piRNA 3′ end recognition and association and may cause structural and functional alterations in PIWIL4. However, among the PIWI family members, PIWIL4 shows the least conservation between humans and mice. The percentage of consensus sequences is 96.6% in PIWIL1, 92.5% in PIWIL2, and only 85.2% in PIWIL4. The binding preference changes for piRNAs induced by this mutation may vary between humans and mice. Based on our transcriptome data, we think that piRNA sequencing may not fully capture the species-specific effects of this mutation. Finally, considering the differences in repetitive DNA elements, the activity of transposable elements and the DNA methylation machinery between mice and humans, species-specific candidate regulatory sequences in repetitive DNA elements may contribute to the observed phenotypic disparity [43] (Figure 4d). It is well-known that deficiencies in piRNA pathway genes lead to different testicular phenotypes between humans and mice [19]. The underlying mechanisms warrant further research.

Although we could not validate the inheritance models due to the unavailability of parental DNA samples, patients carrying heterozygous mutations in PIWIL4 exhibited nonobstructive azoospermia. Although single gene mutations can cause human genetic diseases, these conditions may also result from the combined effects of multiple mutations. Specifically, some heterozygous NOA patients may present with a combination of PIWIL4 mutations and other lesions. Heterozygous mutations in some genes can cause or increase the risk of male infertility such as GALNTL5 in asthenozoospermia [44]; CEP70, UBE2B and ITPR1 in azoospermia [45,46,47]; KLHL10 and PRKAR1A in oligozoospermia [48,49]; SEPT12 in oligoasthenozoospermia or asthenoteratozoospermia [50]; and ODF2 in multiple morphological abnormalities of the sperm flagella (MMAF) [51]. Haploinsufficiency of functional genes or the increased expression of dominant negative variants could cause the phenotypes described above. In our study, homozygous and heterozygous mutant mice showed an impaired expression of retrotransposons during the first wave of spermatogenesis, with the latter exhibiting milder dysregulation. Given the above-mentioned difference between humans and mice, it is possible that the PIWIL4 mutant, with its binding piRNAs, dysregulates some important genes involved in human spermatogenesis.

## 5. Conclusions

In conclusion, our identification of a piRNA-binding mutation in PIWIL4 provides valuable insights into male infertility, highlighting the impact of species-specific piRNA sequences and targeted retrotransposons. The diverse functions of PIWIL4, from de novo methylation in gonocytes to self-renewal in adult spermatogonia, increase the complexity to the observed phenotype. It remains unknown how retrotransposons and L1orf1p expression recover during the steady state of spermatogenesis. Nevertheless, our study expands the possible mechanisms by which human mutations can cause male infertility and lays the foundation for further research into the crucial role of PIWIL4 in both mice and humans.

## Data Availability

The RNA-Seq data of mice testis in this study have been deposited in the Gene Expression Omnibus (GEO) dataset under the accession code GSE258973. All data are available from the corresponding author.

## Supplementary Materials

The following supporting information can be downloaded at: https://www.mdpi.com/article/10.3390/biom15020297/s1, Figure S1: Off-target effect of CRISPR-Cas9 in *Piwil4* knock-in mice; Figure S2: Adult male heterozygous and homozygous knock-in mutation mice exhibited normal sperm morphology and fertility; Figure S3: Heterozygous and homozygous knock-in mutation male mice exhibited normal phenotype during the first wave of spermatogenesis; Figure S4: The piRNA loading activity of PIWIL4 was altered in mutant mice; Figure S5: PIWIL4+ mutant SSCs maintained normal spermatogenesis. Table S1: antibodies and reagents; Original images of Figure 2b, Figure 3a and Figure S2 can be found in supplementary materials.
