# Supplementary material for: Functional Investigation of a Novel PIWIL4 Mutation in Nonobstructive Azoospermia During the First Wave of Spermatogenesis
Source: Biomolecules. 2025 Feb 17;15(2):297. doi: 10.3390/biom15020297 (PMC11852923; doi:10.3390/biom15020297)
Supplement: Supplementary file 1 [file biomolecules-15-00297-s001.zip › Supplemental figures.pdf]

Figure S1: Off-target effect of CRISPR-Cas9 in *Piwil4* knock-in mice

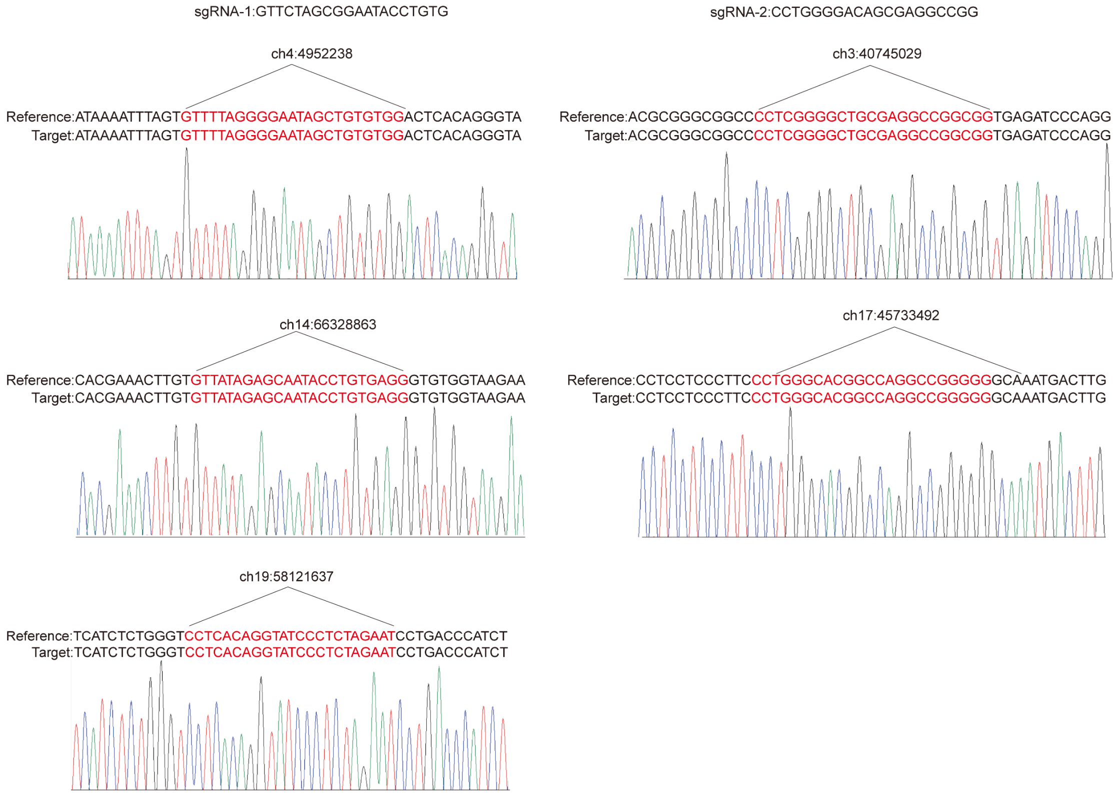

Figure S2: Adult male heterozygous and homozygous knock-in mutation mice exhibited normal sperm morphology and fertility

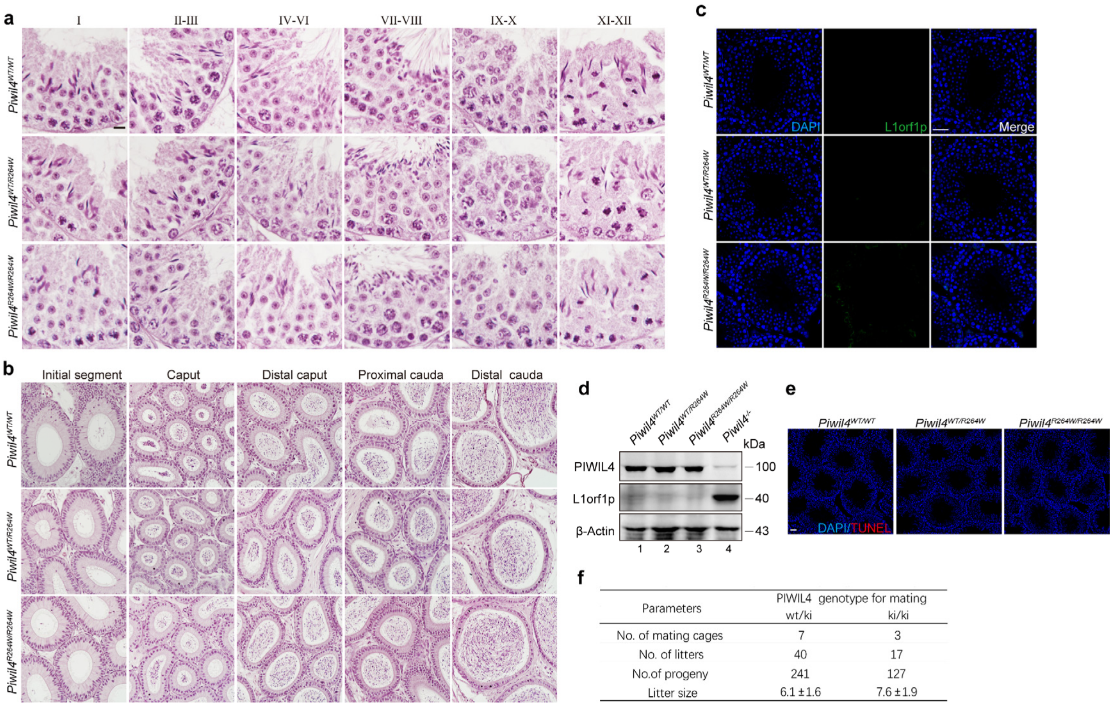

Figure S3: Heterozygous and homozygous knock-in mutation male mice exhibited normal

phenotype during the first wave of spermatogenesis

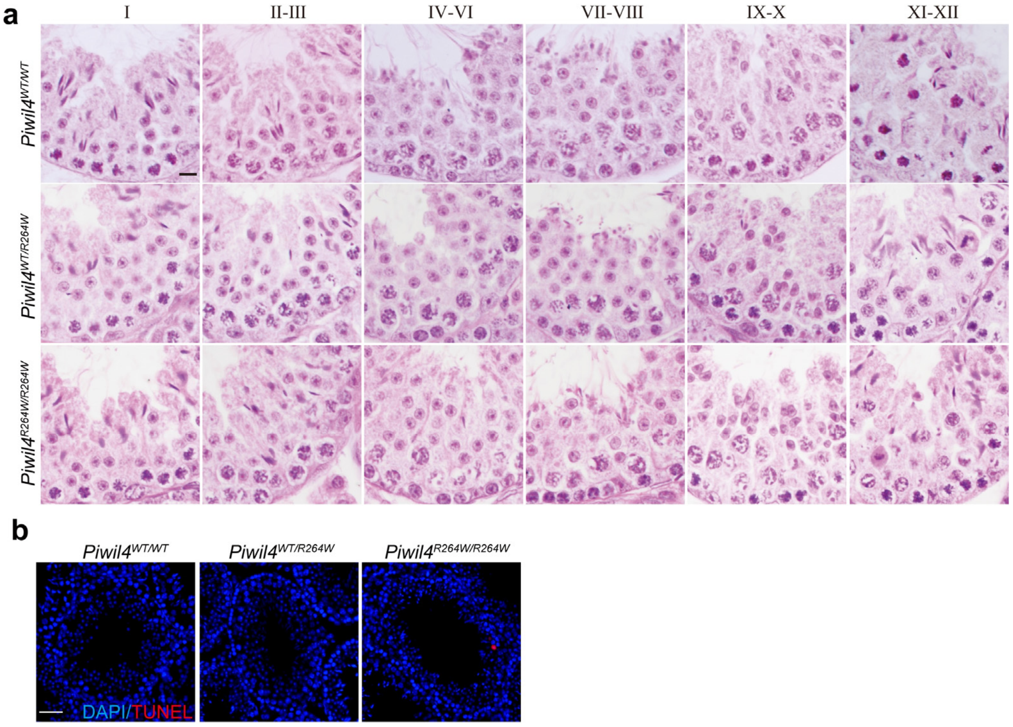

Figure S4: The piRNA loading activity of PIWIL4 was altered in mutant mice

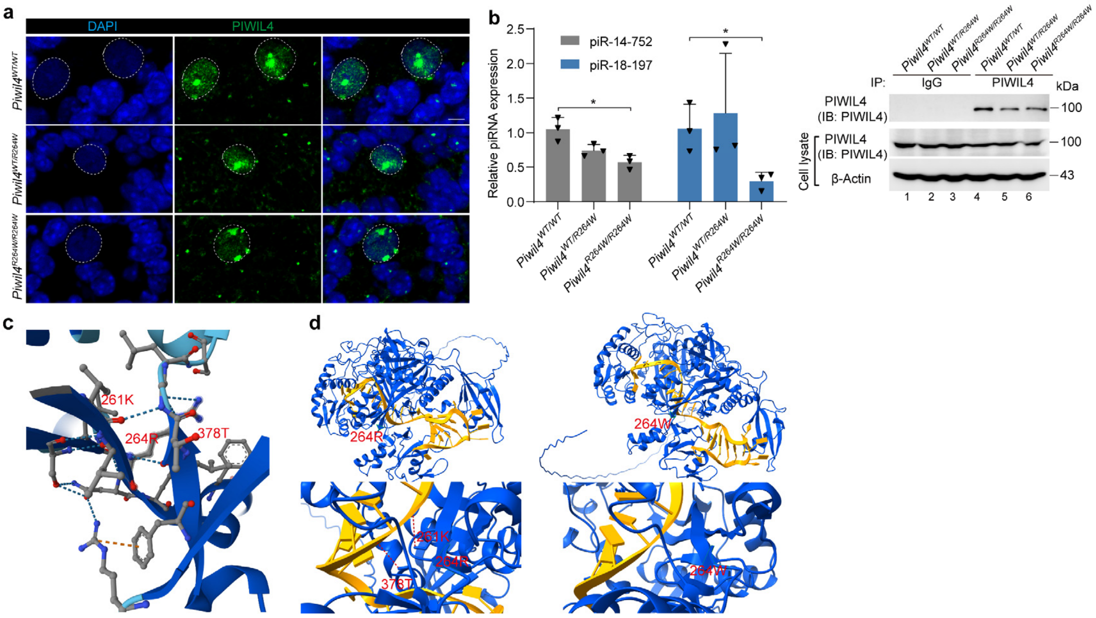

Figure S5: PIWIL4+ mutant SSCs maintained normal spermatogenesis.

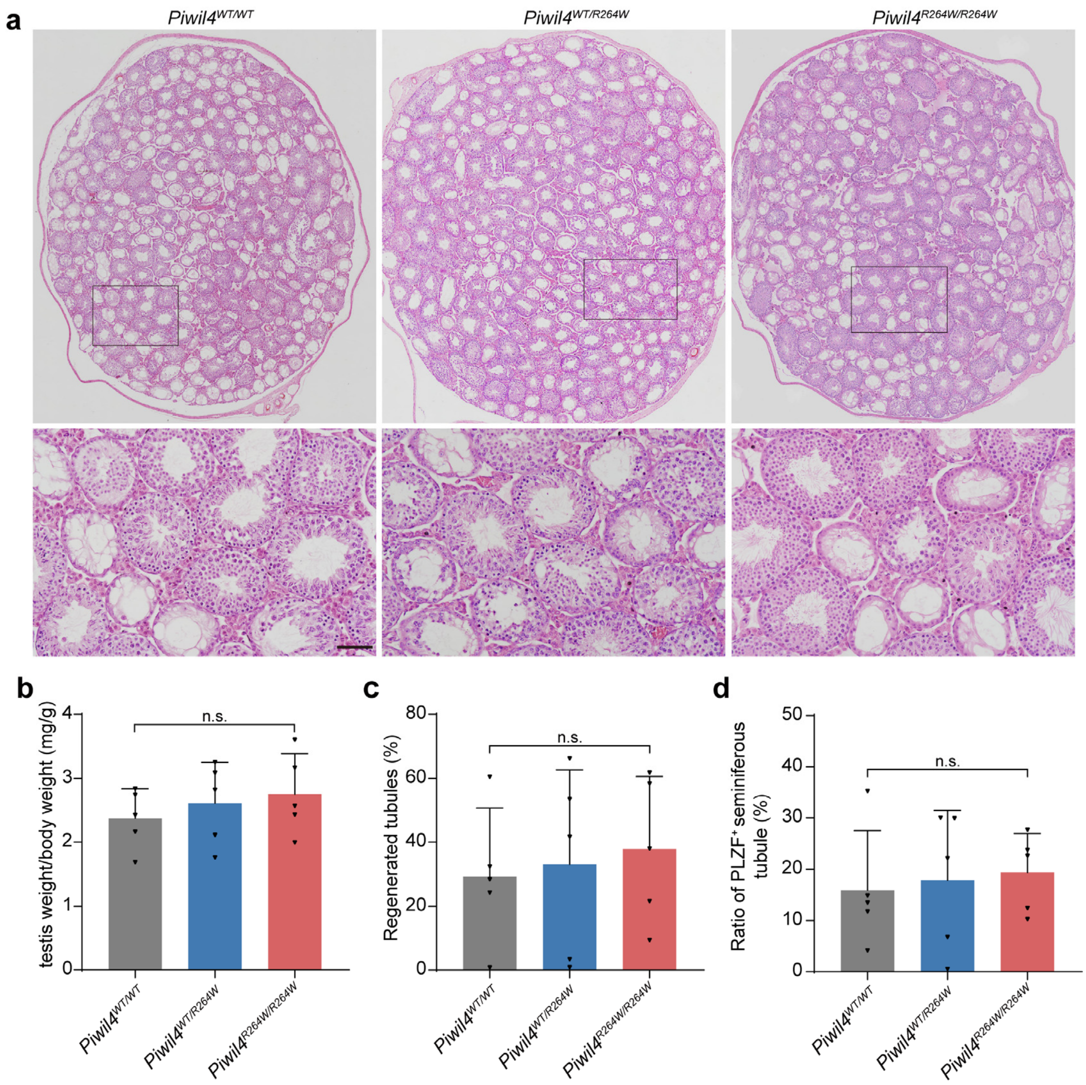
a
Piwil4WT/WT
Piwil4WT/R264W
Piwil4R264W/R264W
b
testis weight/body weight (mg/g)
n.s.
0
1
2
3
4
Piwil4WT/WT
Piwil4WT/R264W
Piwil4R264W/R264W
c
Regenerated tubules (%)
n.s.
0
20
40
60
80
Piwil4WT/WT
Piwil4WT/R264W
Piwil4R264W/R264W
d
Ratio of PLZF+ seminiferous tubule (%)
n.s.
0
10
20
30
40
50
Piwil4WT/WT
Piwil4WT/R264W
Piwil4R264W/R264W
